# Supplementary material for: Identification of photoperiod-induced specific miRNAs in the adrenal glands of Sunite sheep (Ovis aries)
Source: Front Vet Sci. 2022 Jul 22;9:888207. doi: 10.3389/fvets.2022.888207 (PMC9354845; doi:10.3389/fvets.2022.888207)
Supplement: Supplementary file 1 [file Data_Sheet_1.ZIP › Supplementary file/miRDeep2 file.zip!/miRDeep2 file/Know_Structure/SP42A3/oar-mir-199a.pdf]

miRBase precursor : oar-mir-199a
Total read count : 118601
oar-miR-199a-3p read count : 71041
remaining reads : 47560

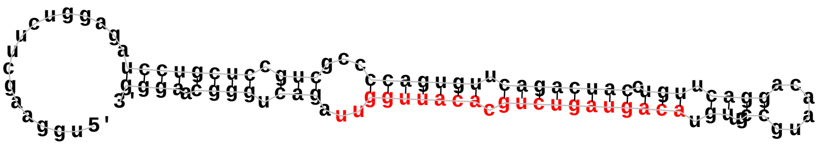

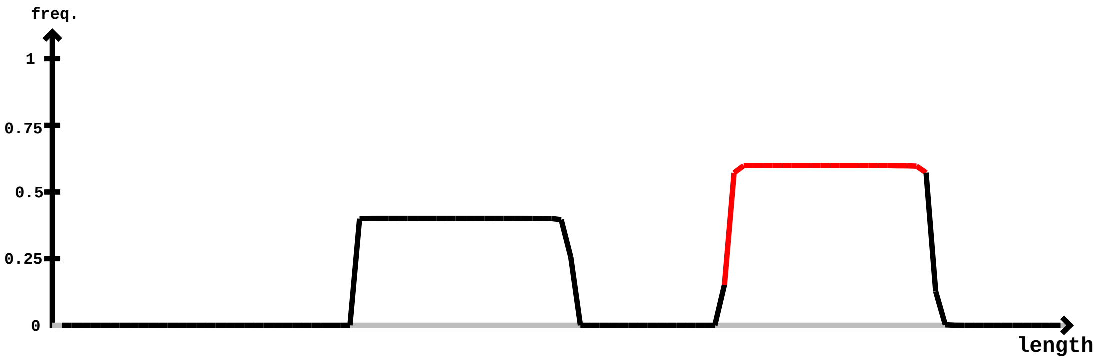


```
                                                                              oar-miR-199a-3p
5'- uggaagcuucuggagauccugcuccgucgccccaguguucagacuaccuguucaggacaaugccguuguacaguagucugcacauugguuagacugggcaaggg   -3'    exp
    .................(((((((.(((...(((((((.((((((((.(((.((((......))..)).))))))))))).)))))))...))).)))).))).   reads   mm   sample
    .............................ccccaguguucagacuaccuguu....................................................   5       0    seq
    .............................ccccaguguucagacuaccuguuc...................................................   4       0    seq
    ..............................cccaguguucagacuacc........................................................   7       0    seq
    ..............................cccaguguucagacuaccu.......................................................   28      0    seq
    ..............................cccaguguucagacuaccug......................................................   33      0    seq
    ..............................cccaguguucagacuaccugu.....................................................   490     0    seq
    ..............................cccaguguucagacuaccuguu....................................................   16662   0    seq
    ..............................cccaguguucagacuaccuguuc...................................................   30202   0    seq
    ..............................cccaguguucagacuaccuguucagg................................................   1       0    seq
    ...............................ccaguguucagacuaccugu.....................................................   3       0    seq
    ...............................ccaguguucagacuaccuguu....................................................   30      0    seq
    ...............................ccaguguucagacuaccuguuc...................................................   83      0    seq
    ................................caguguucagacuaccuguu....................................................   3       0    seq
    ................................caguguucagacuaccuguuc...................................................   8       0    seq
    ....................................................caggacaaugccguuguacagu..............................   1       0    seq
    ....................................................................uacaguagucugcacau...................   1       0    seq
    ....................................................................uacaguagucugcacauu..................   17      0    seq
    ....................................................................uacaguagucugcacauug.................   6       0    seq
    ....................................................................uacaguagucugcacauugg................   51      0    seq
    ....................................................................uacaguagucugcacauuggu...............   1555    0    seq
    ....................................................................uacaguagucugcacauugguu..............   14933   0    seq
    ....................................................................uacaguagucugcacauugguua.............   1453    0    seq
    ....................................................................uacaguagucugcacauugguuag............   17      0    seq
    ....................................................................uacaguagucugcacauugguuaga...........   2       0    seq
    .....................................................................acaAuagucugcacau...................   1       1    seq
    .....................................................................acaguagucugcacauu..................   53      0    seq
    .....................................................................acaguagucugcacauug.................   18      0    seq
    .....................................................................acaguagucugcacauugg................   70      0    seq
    .....................................................................acaguagucugcacauuggu...............   1256    0    seq
    .....................................................................acaguagucugcacauugguu..............   35667   0    seq
    .....................................................................acaguagucugcacauugguua.............   12503   0    seq
    .....................................................................acaguagucugcacauugguuag............   164     0    seq
    .....................................................................acaguagucugcacauugguuaga...........   32      0    seq
    .....................................................................acaguagucugcacauugguuagG...........   1       1    seq
    ......................................................................caguagucugcacauugg................   3       0    seq
```

oar-miR-199a-3p

```
uggaagcuucuggagauccugcuccgucgccccaguguucagacuaccuguucaggacaaugccguuguacaguagucugcacauugguuagacugggcaaggg
......................................................................caguagucugcacauuggu...............     108     0     seq
......................................................................caguagucugcacauugguu..............     2207    0     seq
......................................................................caguagucugcacauugguua.............     885     0     seq
......................................................................caguagucugcacauugguuag............     17      0     seq
......................................................................caguagucugcacauugguuaga...........     5       0     seq
.......................................................................aguagucugcacauugguu..............     11      0     seq
.......................................................................aguagucugcacauugguua.............     5       0     seq
```
